# A multi-centre randomised double-blind placebo-controlled trial to evaluate the value of a single bolus intravenous alfentanil in CT colonography

**DOI:** 10.1186/1471-230X-13-94

**Published:** 2013-05-25

**Authors:** Thierry N Boellaard, Marije P van der Paardt, Markus W Hollmann, Susanne Eberl, Jan Peringa, Lex J Schouten, Giedre Kavaliauskiene, Jurgen H Runge, Jeroen AW Tielbeek, Jaap Stoker

**Affiliations:** 1 Department of Radiology, Academic Medical Center, University of Amsterdam, Meibergdreef 9, Amsterdam, 1100 DD, the Netherlands; 2 Department of Anaesthesiology, Academic Medical Center, University of Amsterdam, Amsterdam 1100 DD, the Netherlands; 3 Department of Radiology, Onze Lieve Vrouwe Gasthuis, Amsterdam 1091 AC, the Netherlands

**Keywords:** Randomised controlled trial, Colonography, Computed tomographic, Alfentanil, Analgesics, Opioid, Patient satisfaction

## Abstract

**Background:**

Pain is common during colonic insufflation required for CT colonography. We therefore evaluate whether a single intravenous alfentanil bolus has a clinically relevant analgesic effect compared with placebo in patients undergoing CT colonography.

**Methods:**

A prospective multi-centre randomised double-blind placebo-controlled trial was performed in patients scheduled for elective CT colonography. Patients were randomised to receive either a bolus of 7.5 μg/kg alfentanil (n = 45) or placebo (n = 45). The primary outcome was the difference in maximum pain during colonic insufflation on an 11-point numeric rating scale. We defined a clinically relevant effect as a maximum pain reduction of at least 1.3 points. Secondary outcomes included total pain and burden of CT colonography (5-point scale), the most burdensome aspect and side effects. Our primary outcome was tested using a one-sided independent samples t-test.

**Results:**

Maximum pain scores during insufflation were lower with alfentanil as compared with placebo, 5.3 versus 3.0 (*P* < 0.001). Total CT colonography pain and burden were also lower with alfentanil (2.0 vs. 1.6; *P* = 0.014 and 2.1 vs. 1.7; *P* = 0.007, respectively). With alfentanil fewer patients rated the insufflation as most burdensome aspect (56.1% vs. 18.6%; *P* = 0.001). Episodes with desaturations < 90% SpO2 were more common with alfentanil (8.1% vs. 44.4%; *P* < 0.001, but no clinically relevant desaturations occurred.

**Conclusions:**

A low-dose intravenous alfentanil bolus provides a clinically relevant reduction of maximum pain during CT colonography and may improve the CT colonography acceptance, especially for patients with a low pain threshold.

**Trial registration:**

Dutch Trial Register: NTR2902

## Background

Computed tomographic (CT) colonography is an accurate technique for the detection of colorectal cancer and clinically relevant polyps and it is a less invasive alternative for colonoscopy [1-7]. In clinical practice CT colonography is widely used and it has been adopted as colorectal cancer screening tool in the United States and is considered for screening in other countries [8,9].

Sufficient colonic distension is mandatory for visualisation of the bowel wall [10], but insufflation causes the bowel to stretch and may result in painful colonic cramps [11-16]. Colonic insufflation is one of the most burdensome aspects of CT colonography [13-15]. In several studies the pain and burden scores of CT colonography even compare unfavourably with conventional colonoscopy under conscious sedation [11,12,15,16], although in other studies CT colonography is favoured over colonoscopy [3,17]. However discomfort, pain and anxiety (including possible side effects of anxiety [18]) are detrimental for acceptance of the test, both in clinical practice as in screening.

During conventional colonoscopy administration of analgesics is regular practice. To the best of our knowledge, no analgesics are administered during CT colonography and no studies have evaluated this option. To induce sufficient analgesia for acute pain during CT colonography an opioid is most suitable because of the analgesic potency [19-21]. Hereby intravenous administration allows more precise timing of the peak effect compared with oral administration.

In sigmoidoscopy, a fentanyl bolus has been shown to improve pain scores [22]. Because of the CT colonography procedure time is approximately 20 minutes [23] an opioid with a rapid onset and short elimination time would be suitable and prevents long recovery times. Alfentanil is such a short-acting opioid (maximum effect within 1–2 minutes and subsequent distribution half life values of 1 and 14 minutes) [24]. The need for recovery facilities could have negative consequences for its widespread clinical use for CT colonography and in particular in screening [25].

Before considering the use of opioids during CT colonography, it is necessary to demonstrate a clinically relevant pain reduction, beneficial effect on the burden and acceptance, without detrimental effects on safety, procedure time and recovery time.

We hypothesised that a single bolus intravenous alfentanil will give a clinically relevant reduction in maximum pain defined as at least 1.3 point reduction on an 11-point numeric rating scale [26-28].

## Methods

### Design

**Figure 1:**
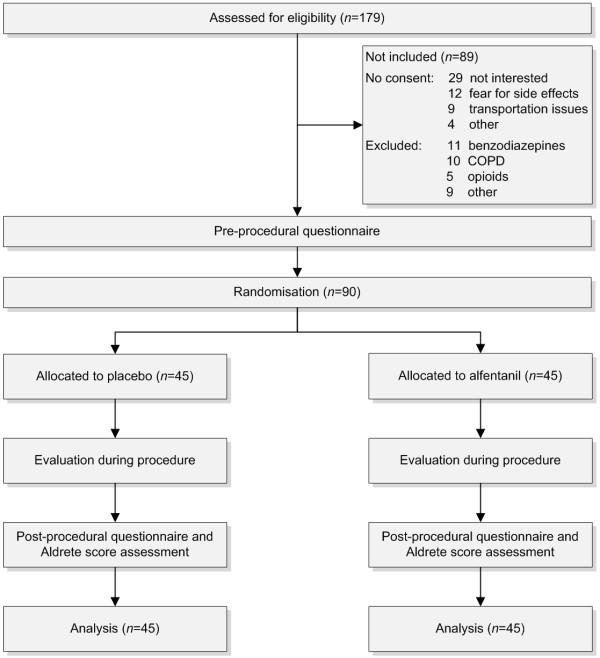
Flow chart of the study.

We performed a prospective, multi-centre, randomised, double-blind, placebo-controlled trial to evaluate whether a single bolus intravenous alfentanil has a clinically relevant analgesic effect in patients scheduled for elective CT colonography. We evaluated possible differences between alfentanil and placebo at different time points (Figure 1). This study was approved by the institutional review board of the Academic Medical Center and all participants gave their written informed consent. The study protocol has previously been described in detail [29] and the trial was registered in the Dutch Trial Register: NTR2902. The study was conducted in accordance with the protocol and in compliance with the moral, ethical, and scientific principles governing clinical research as set out in the Declaration of Helsinki (1989) and Good Clinical Practice (GCP). The CONSORT 2010 Statement was used as guide for our reporting [30].

### Outcomes measures

The primary outcome was the difference in maximum pain score between patients receiving alfentanil compared with placebo. We had defined a clinically relevant effect as a pain reduction of at least 1.3 point on an 11-point numeric rating scale, as this is considered the minimally important difference using this scale [26-28].

Secondary outcome measures were differences between patients receiving alfentanil and placebo regarding: pain scores per insufflation position, pain and burden of the CT colonography procedure and individual CT colonography aspects (e.g. insufflation, bowel preparation etc.), side effects, vital parameters, procedure time and recovery time.

### Population

Consecutive patients aged 18–85 years and scheduled for elective CT colonography were assessed for eligibility in two institutions in Amsterdam, the Netherlands: Academic Medical Center, University of Amsterdam (academic institution) and Onze Lieve Vrouwe Gasthuis (teaching hospital). Patients were assessed for eligibility by telephone by one of the research physicians (T.N.B., M.P.P. or L.J.S.). CT colonography was performed in symptomatic and surveillance patients only. We used the following exclusion criteria: heart rate < 50 beats per minute; systolic blood pressure < 90 mmHg; severe chronic obstructive pulmonary disease; liver disease defined as a Child-Pugh score of > 4; alfentanil allergy; pregnancy; increased intracranial pressure; use of MAO-inhibitors within two weeks before CT colonography; use of barbiturates, opiates or daily benzodiazepine.

### Power calculation

We powered to detect a difference in maximum pain score between the placebo and alfentanil group, using nQuery Advisor 7.0 (Statistical Solutions Ltd., Cork, Ireland). Based on our anaesthesiologists (S.E. and M.W.H.) experience a maximum pain reduction of 1.5 points was expected. A previously performed population screening trial served as pilot data to assess distribution and standard deviation [15]. We calculated for a one-sided t-test, 1.5 point difference, 80% power, 0.05 α error and a standard deviation of 2.6. We assumed 5% withdrawal and therefore our calculation resulted in groups of 45 patients.

### Intervention

Two research physicians (J.H.R. and M.C.H.) generated a randomisation list using nQuery. The list was kept by three research physicians (J.H.R., M.C.H. and J.A.W.T.) not involved in the patient recruitment, CT colonography procedure or data collection. Included patients were randomised into two groups of 45 patients (1:1 ratio and blocks of six). Group 1 received a single bolus of 7.5 μg/kg actual body weight alfentanil (Rapifen, Janssen-Cilag, Tilburg, the Netherlands). Group 2 received 0.9% saline solution as placebo. Both groups received 0.075 mL/kg actual body weight fluid. Study medication was prepared by the physicians who kept the randomisation list. The medication ampule was placed in a signed sealed envelope near the CT scanner room to allow deblinding in case of a medical emergency. The study medication was administered (double-blind) through a 20 Gauge intravenous cannula, 1½ minute following administration of a spasmolytic agent. After the procedure one of the physicians who kept the randomisation list collected the envelope.

### CT colonography

We used a 24-hour preparation with low-fibre diet and three or four bottles 50 mL iodinated contrast, meglumine ioxithalamate (Telebrix, Guerbet, Aulnay sous Bois, France) [31,32]. For bowel relaxation 1 mL (20 mg) butylscopalamine bromide (Buscopan, Boehringer-Ingelheim, Ingelheim, Germany) or, if contraindicated 1 mL (1 mg) glucagon (GlucaGen, Novo Nordisk A’S, Bagsvaerd, Denmark) was used [33,34]. An automated carbon dioxide insufflator (PROTOCO2L, Bracco, EZEM, Lake Success, USA) and a flexible 20 French rectal catheter were used with insufflation in three positions: right decubitus, supine and left decubitus position. We aimed for three litres insufflation (1.3, 0.8 and 0.9 litres per position, respectively). The insufflation pressure was gently increased during insufflation (maximum pressure of the insufflator is 25 mmHg) and set on 20 mmHg when the target volume of three litres was met or after five minutes of insufflation irrespective of the target volume. Subsequently scan acquisitions were performed in prone and supine position and intravenous contrast medium iopromide (Ultravist 300, Bayer B.V., Mijdrecht, the Netherlands) was given in case of clinical suspicion for colorectal cancer. Prone position was the first acquisition position when intravenous contrast was used and the second scan position when only unenhanced acquisitions were performed.

### Evaluation during procedure

Pain scores were assessed with an 11-point numeric rating scale at the end of prone scan acquisition position and for all three insufflation positions (0 = no pain and 10 = worst pain imaginable).

During the procedure, patients’ vital parameters were monitored (PM50, Contec Medical Systems CO, LTD, Qinhuangdao, China). We registered values at standard time points: before spasmolytic injection, 1½ minutes after spasmolytic injection and at 5 and 10 minutes after starting the alfentanil injection. The value 1½ minute after spasmolytic injection was the reference value for comparisons with values after study medication injection, because the spasmolytic can influence the vital parameters [35]. Heart rate and oxygen saturation were measured continuously with the same device and stored measurements were analysed afterwards. Furthermore all side effects and the time in the scanner room were recorded.

### Monitoring

**Figure 2:**
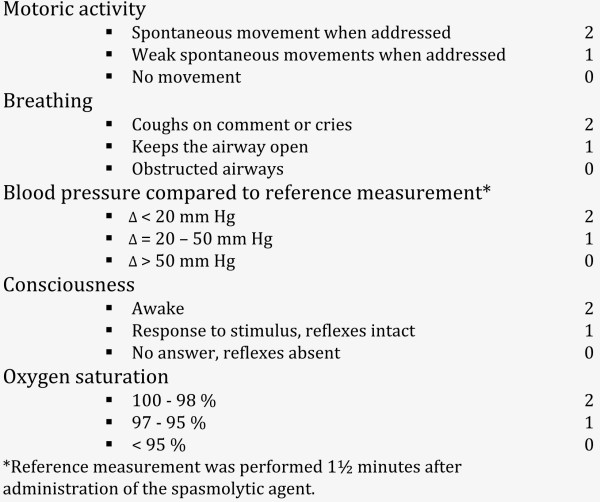
**Modified Aldrete score **[38]. The modified Adrete score was assessed at three time points: at arrival in the waiting room after the procedure, and at 30 and 60 minutes after study medication injection. The maximum Adrete score is 10.

To evaluate recovery after the procedure the Aldrete score, a commonly used recovery score [36,37], was assessed at arrival in the waiting room and at 30 and 60 minutes after administration of the study medication (Figure 2) [38].

### Questionnaires

Baseline characteristics, procedure expectations and procedure experience were assessed using a pre- and post-procedural questionnaire and largely originated from a previous study [15]. The pre-procedural questionnaire consisted of 22 questions and was filled out before randomisation and the post-procedural questionnaire consisted of 17 questions and was filled out 30 minutes after the procedure. Questions about expectations included burden and pain of the bowel preparation, intravenous cannula insertion, bowel insufflation, and the total procedure on a standard formatted 5-point scale (not at all, slight, some, rather, and extremely). Identical questions on experience were asked using the same standard formatted 5-point scale. Further, the most painful or burdensome aspect was assessed and whether they would accept CT colonography as a screening test.

### Follow-up

All patient symptoms reported to their general practitioner up to one month after the procedure were assessed for their likelihood of being related to alfentanil by two blinded anaesthesiologists (S.E. and M.W.H.).

### Image evaluation

Two independent observers (T.N.B. and G.K., both evaluated > 250 CT- colonographies) evaluated all CT images for distension, collapse, diagnostic adequacy and diverticulosis [39]. Evaluation was done for six segments separately and distension and collapse was also evaluated for prone and supine separately. Bowel distension was scored on a 4-point scale based on the worst part of the segment (0-25%, 25-50%, 50-75% and 75-100% distension) [40]. The presence of collapse was scored (yes or no). To assess diagnostic adequacy, observers scored whether detection of ≥ 6 mm lesions was possible based on the distension of prone and supine position combined (yes or no). Diverticulosis was scored on a 4-point scale [41].

### Statistics

All calculations were performed using SPSS version 18.0 (SPSS inc., Chicago, Illinois, USA) and a *P* value of < 0.05 indicated a statistically significant difference. All data entry was completed before deblinding. For the difference in maximum pain score during insufflation, we used a one-sided independent samples t-test. Univariate analyses were performed with linear regression to identify possible confounders. The four most influential variables with a *P* < 0.1 would be included in a multivariate analysis.

For time calculations and pain scores per position we used a two-sided independent t-test. Differences between categorical values were determined using a Chi-square test and binary values with the Fishers exact test. A (weighted) kappa was calculated to assess interobserver agreement with regards to distension, collapse, diagnostic adequacy and diverticulosis scores. We used the first observer scores to perform ordered regression for distension scores. Variables with *P* < 0.1 in univariable analysis were added to the regression analysis as confounder (diverticulosis, BMI and spasmolytic were tested). Logistic regression was used for collapse and diagnostic adequacy scores.

## Results

Between May 2011 and June 2012 a total of 179 patients were screened for eligibility. We included 90 patients, 54 did not consent and 35 were excluded (Figure 1). Baseline characteristics were similar for both groups (Table 1). All participants filled out pre- and post- procedural questionnaires and none of the patients was lost to follow-up.

**Table 1:** Baseline patient characteristics

**Characteristic**	**Placebo (n = 45)**	**Alfentanilb (n = 45)**	***P *value**
Male (%)	44.4 (20/45)	31.1 (14/45)	0.28
Age (years)	62.2	62.7	0.86
Weight (kg)	75.5	76.3	0.83
Height (cm)	172	170	0.41
BMI (kg/m2)	25.6	26.2	0.59
Education (%)			0.83
primary	6.7 (3/45)	6.7 (3/45)	
secondary	53.3 (24/45)	46.7 (21/45)	
tertiary	40.0 (18/45)	46.7 (21/45)	
Ethnicity (%)			0.90
Dutch	93.3 (42/45)	91.1 (41/45)	
Surinam	4.4 (2/45)	6.7 (3/45)	
other	2.2 (1/45)	2.2 (1/45)	
First CT colonography (%)	100 (45/45)	100 (45/45)	1.00
Indication abdominal pain (%)	29.5 (13/44)	15.9 (7/44)	0.20
Spasmolytic (%):			0.60
buscopan	86.7 (39/45)	84.4 (38/45)	
glucagon	13.3 (6/45)	13.3 (6/45)	
no	0 (0/45)	2.2 (1/45)	
Oxygen saturation (%SpO2)	98	98	0.47
Heart rate (b/m)	77.8	79.0	0.70
Systolic blood pressure (mmHg)	155	158	0.59
Diastolic blood pressure (mmHg)	86	89	0.28
Expected burden (1–5)	3	3	0.31
Expected pain (1–5)	3	3	0.46

This table shows mean values, unless indicated otherwise; medians are presented for oxygen saturation, expected pain and burden.

For all percentage the numbers are given between brackets. No significant differences were present between baseline characteristics of the two groups.

### Evaluation during procedure

Maximum insufflation pain in the alfentanil group was 2.3 (95%CI 1.3-3.4) point lower on the 11-point numeric rating scale compared with the placebo group (Table 2). All possible confounders were equally distributed over the groups and all possible confounders had a *P* > 0.1 in the univariate analysis. Therefore we did not perform a multivariate analysis or correct for confounders.

**Table 2:** Procedural characteristics

**Outcome**	**Placebo (n = 45)**	**Alfentanil (n = 45)**	***P *value**
Maximum pain score	5.3 (2.5)	3.0 (2.5)	<0.001
Pain score			
1 right decubitus	1.8 (2.4)	0.4 (1.0)	<0.001
2 supine	3.4 (2.6)	1.4 (2.0)	<0.001
3 left decubitus	5.1 (2.5)	2.6 (2.2)	<0.001
4 prone	3.0 (2.6)	1.6 (2.5)	0.01
Volume CO2 insufflation (litre)	4.6 (1.4)	4.7 (1.2)	0.99
Pressure at end insufflation (mmHg)	22 (20–25)	22 (19–25)	0.81
Procedural time (minutes)	24.4 (4.2)	24.8 (4.7)	0.64

This table provides mean values with the standard deviation between brackets. Except the pressure at the end of insufflation which is a median value with interquartile range.

Pain scores 1, 2 and 3 were asked before performing the scan acquisitions. Pain score 4 was asked with the patient in the prone scan position. No pain score was asked during the supine scan position.

Significant differences in pain score between the alfentanil group and placebo group were also present for all insufflation positions separately. The amount of medication given, litres insufflation, pressure at the end of insufflation and procedural time were similar (Table 2).

Side effects during CT colonography are summarised in Table 3. Only dizziness and desaturations (SpO_2_ < 90% of any duration) were significantly more common in the alfentanil group. All desaturations resolved spontaneously and none of the side effects interfered with the procedure.

**Table 3:** Side effects

	**Placebo**	**Alfentanil**	***P *value**
Nausea	2.2 (1/45)	6.7 (3/45)	0.62
Vomiting	0.0 (0/45)	2.2 (1/45)	1.00
Dry mouth	2.2 (1/45)	0.0 (0/45)	1.00
Blurry vision	4.4 (2/45)	2.2 (1/45)	1.00
Dizziness	4.4 (2/45)	37.8 (17/45)	<0.001
Sleepy	0.0 (0/45)	2.2 (1/45)	1.00
Dyspnoeic	0.0 (0/45)	2.2 (1/45)	1.00
Sweating	0.0 (0/45)	2.2 (1/45)	1.00
Pressure sensation	0.0 (0/45)	2.2 (1/45)	1.00
Desaturation (SpO2 < 90%) *	8.1 (3/37)	44.4 (16/36)	<0.001
Desaturation >15 seconds (SpO2 < 90%)	8.1 (3/37)	19.4 (7/36)	0.19
Desaturation (SpO2 < 85%) #	2.7 (1/37)	16.7 (6/36)	0.06
Desaturation >15 seconds (SpO2 < 85%) #	0.0 (0/37)	0.0 (0/36)	1.00
Irregular heart rhythm	18.9 (7/37)	16.7 (6/36)	1.00
Systolic blood pressure at 5 minutes (20% decrease)	8.9 (4/45)	11.1 (5/45)	1.00
Diastolic blood pressure at 5 minutes (20% decrease)	20.0 (9/45)	15.6 (7/45)	0.78
Heart rate at 5 minutes (20% decrease)	4.4 (2/45)	0.0 (0/45)	0.49

This table shows the percentage of patients with different side effects for the placebo and alfentanil group.

* All > 15 sec and < 85% desaturations are also counted for this category.

# All < 85% saturations lasted 2–9 seconds, on average 4.2 seconds.

### Experience questionnaire

**Figure 3:**
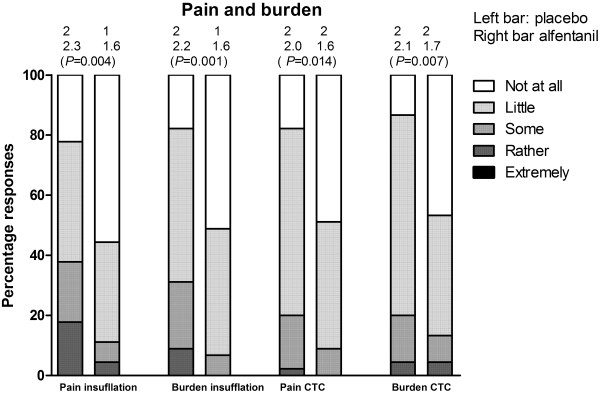
**Distribution of pain and burden scores.** This figure shows the distribution of pain and burden scores (5-point scale) of colonic insufflation and the total CT colonography procedure. Above the bars the medians, means and *P* values are presented (from top to bottom). Pain and burden of insufflation and complete CT colonography procedure were significantly lower with alfentanil.

Median pain and burden of intravenous cannula insertion were 1 (IQR 1–2) for both groups combined. Median pain and burden of the bowel preparation were 1 (IQR 1–1.25) and 2 (IQR 1–3) for both groups combined. Other experience questionnaire outcomes are reported in Table 4. Pain and burden of the insufflation procedure and total CT colonography procedure were in favour of the alfentanil group (Figure 3).

**Table 4:** Experience questionnaire

**Outcome**	**Placebo**	**Alfentanil**	***P *value**
Average insufflation pain	3.8 (2.1)	1.8 (2.0)	<0.001
Insufflation most burdensome aspect	56.1% (23/41)	18.6% (8/43)	0.001
Insufflation most painful aspect	60.0% (24/40)	34.1% (15/44)	0.053
Advise for screening	92.7% (38/41)	100% (44/44)	0.11

This table shows the mean insufflation pain with standard deviation between brackets.

For the other outcomes the percentages are provided with the numbers between brackets.

### Monitoring and recovery

**Figure 4:**
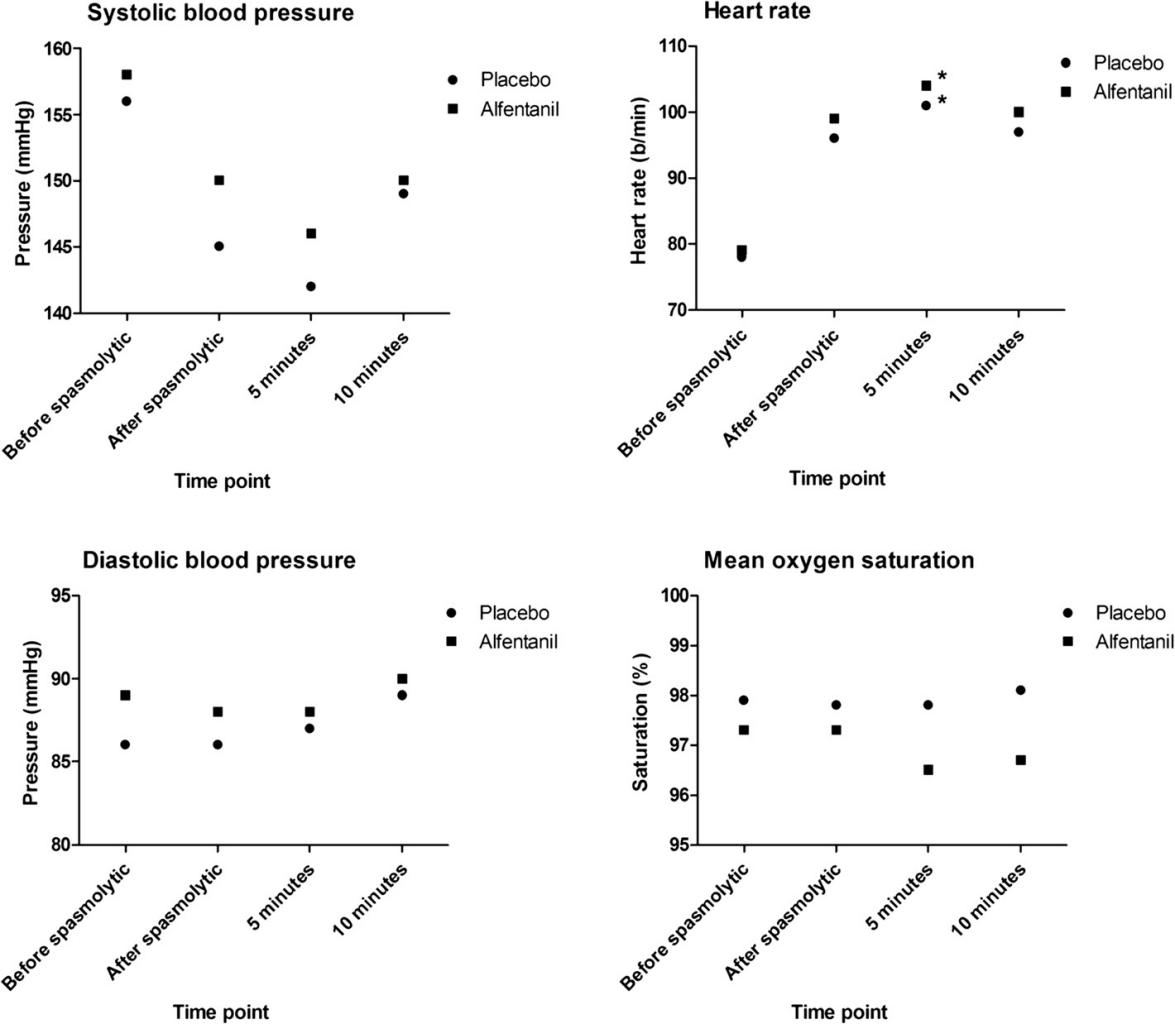
**Vital parameters.** Systolic and diastolic blood pressure, the heart rate and saturation over time for the alfentanil and placebo group. * indicates a significant difference.Statistical differences were calculated between the reference measurement 1 ½ minutes after spasmolytic and 5/10 minutes after alfentanil injection. The heart rate was significantly higher after 5 minutes for both placebo and alfentanil group. No other significant differences were observed.

Vital parameters are shown in Figure 4. For both groups the only significant difference was found between heart rate baseline measurement 1½ minute after spasmolytic injection and 5 minutes after study medication injection. As shown in Table 5, the Aldrete score was only significantly lower for the alfentanil group at arrival in the waiting room.

**Table 5:** Median aldrete scores

**Time point**	**Placebo**	**Alfentanil**	***P *value**
Arrival	10 (9–10)	9 (9–10)	0.047
30 minutes	10 (9–10)	9 (9–10)	0.16
60 minutes	10 (9–10)	9 (9–10)	0.10

This table shows median values with interquartile ranges of the Aldrete scores at arrival, 30 minutes and 60 minutes after study medication injection.

Eight was the lowest Aldrete score in both groups.

### Colonic distension

For all segments and both positions combined no correlation was seen between distension scores and randomisation group (BMI and diverticulosis were confounders) (*P* = 0.41). Additionally no difference was found for supine (*P* = 0.60) and prone (*P* = 0.54) separately. Alfentanil did not influence the total number of collapsed segments (*P* = 0.25), nor the diagnostic adequacy (*P* = 0.15). Interobserver agreement was good for distension and diagnostic adequacy (kappa value 0.62 and 0.65). Interobserver agreement was very good for collapse and diverticulosis, both 0.81.

### Follow-up

Six patients had reported symptoms to the general practitioner in the month after the CT colonography. Three of these were in the alfentanil group. Two of the complaints were rated as possibly related to alfentanil (i.e. constipation and dysuria).

## Discussion

A single bolus 7.5 μg/kg intravenous alfentanil results in a clinically relevant reduction in maximum pain during colonic insufflation required for CT colonography. Importantly, alfentanil also reduced the total pain and burden of the complete CT colonography procedure. Alfentanil did not influence the procedure time and with alfentanil fewer patients considered colonic insufflation the most burdensome aspect of CT colonography. Dizziness and desaturations were the most common side effects of alfentanil, though recovery times were short.

The reduction of maximum pain was more than the 1.3 points on an 11-point numeric rating scale as we hypothesised and which is considered the minimum clinically relevant difference [26-28]. For this scale, a pain score reduction of 2–2.4 points or 33-35% may be of even greater clinical importance [26,27,42]. Both these criteria are also met with the reduction we observed. Pain scores during the prone scan acquisition position was 3.0 in the placebo group and thus lower than during the left decubitus position, likely due to decreased pressure after initial insufflation or habituation to the insufflated colon [41].

Importantly, also the pain and burden of the total CT colonography procedure were reduced. The effect of alfentanil was more evident on the most burdensome aspect, than on the most painful aspect. This is likely because patients experience the bowel preparation as burdensome, but not as painful. With alfentanil, the insufflation becomes less burdensome and therefore the burden of the bowel preparation becomes relatively more important.

The observed dizziness and desaturations are known side effects of alfentanil. The desaturation in the placebo group may indicate that some patients experience spontaneous desaturations during the day. Importantly, all desaturations were not clinically relevant, because they were short and self-limiting we did not had to perform any intervention.

Although we found desaturations with alfentanil, we did not find a SpO2 reduction at 5 and 10 minutes after alfentanil injection. Conti et al. observed a significant saturation reduction with a 10 μg/kg bolus intravenous alfentanil in ASA 1 patients during minor surgery or endoscopy [43].

In colonoscopy opioids are commonly used in combination with a benzodiazepine to induce amnesia. We did not consider this mandatory for CT colonography while this combination leads to a greater respiratory depressant effect than opioids only. The benzodiazepine-induced drowsiness may complicate the CT colonography procedure and recovery facilities may be required.

A number of limitations have to be acknowledged. The dizziness caused by alfentanil may have partly affected the double blind character of the trial. We had anticipated this however, to our knowledge no substance is available that causes dizziness in an equal number of patients as alfentanil and that does not affect the outcomes. We chose to use 0.9% saline solution for the placebo group because it was the solvent for alfentanil and the viscosity and colour was similar to that of alfentanil. Importantly, patients were not aware that dizziness would be more likely related to alfentanil administration than placebo. Pain was assessed in prone scan acquisition position only, as we wanted to limit the number of questions and the pressure is higher in prone position [41]. The time of prone scan acquisition differed some minutes between studies with and without intravenous contrast medium. The influence of prone score on the maximum pain was negligible as these were much lower compared with left decubitus. We chose to use an 11-point numeric rating scale, which is a commonly used scale [26,44]. The visual analogue scale [44] is also commonly used, however this scale is less practical during colonic insufflation on a narrow table, while having colonic cramps and being monitored. Additionally, we have experience with the 11-point numeric rating scale during CT colonography. For the Aldrete score, we chose as reference values blood pressure and heart rate measurements, recorded 1½ minute after injection of the spasmolytic agent. Most patients received butylscopalamine bromide, which increases the heart rate [35]. As the effect of the spasmolytic decreases over time, the heart rate also decreases. Furthermore, most patients are nervous at the beginning of a medical procedure and calm down in the course of the procedure. Both factors might have influenced Aldrete score negatively, although all patients had a normal heart rate and blood pressure after the procedure. Despite the fact the side effects of alfentanil were of minor clinical relevance and the benefit-risk ratio seems to favour alfentanil, a safety profile cannot be made based on 45 patients. Although other studies also have shown safe use of a single bolus low-dose alfentanil [43,45], more data on patient safety is warranted.

When alfentanil is used it is important to realise that monitoring and airway intervention equipment and sufficient knowledge about the pharmacology of opioids and airway interventions should be present. This means that the attendance of a physician is required. For institutions were a technician is performing the CT colonography procedure, adjustments will have to be made in the procedure. All patients receiving intravenous alfentanil require an intravenous cannula, so emergency medication can be given. The above mentioned issues may lead to an increase in costs. Furthermore, when using alfentanil the patients need to arrange transportation, because driving after alfentanil injection is not allowed for 12–24 hours minimum; this can be a large hurdle for implementation of alfentanil [24]. Patients who receive butylscopalamine bromide during CT colonography are already advised not to drive just after the procedure, as this may affect the ability to drive. Because of disadvantages such as the inability to drive, the lack of analgesia has been mentioned as one of the advantages of CT colonography [46,47].

## Conclusions

To the best of our knowledge we are the first to study the use of analgesia for CT colonography. Our study shows that alfentanil provides a clinically relevant reduction in maximum pain in CT colonography, reduces total procedural pain and burden, without detrimental effects on procedural time, recovery time and patient safety. Although desaturations were frequently observed with alfentanil, these were not considered clinically relevant because they were all short-lasting and self-limiting. Therefore alfentanil may well be an option to improve acceptance, although it may impact the logistics associated CT colonography examinations. Now that we know the advantage of alfentanil it can be weighed against the practical hurdles, side effects and costs. Especially for patients with a low pain threshold, a low-dose intravenous alfentanil bolus may be viable option. Our secondary outcomes such as the total procedural pain and burden, the effect on procedure time and recovery time should be confirmed in for these outcomes appropriately powered studies. Furthermore, additional data on a low-dose intravenous bolus injection alfentanil during CT colonography is required to further assess the safety profile.

## Competing interests

The authors declare that they have no competing interests.

## Authors’ contributions

TNB is responsible for drafting the manuscript. TNB, MPP, SE, MWH, and JS are responsible for the study design. MPP, SE, MWH, GK, JP, LJS, JAWT, JHR and JS are responsible for revising the manuscript. TNB, MPP, GK, JP, LJS, JAWT, JHR are responsible for the data acquisition and TNB for the analysis. All authors have read and approved the manuscript.

## Pre-publication history

The pre-publication history for this paper can be accessed here:

http://www.biomedcentral.com/1471-230X/13/94/prepub
